# Massive Femoral Osteolysis Secondary to Loosening of a Cemented Roughened Long Stem: A Case Report

**DOI:** 10.1155/2014/840267

**Published:** 2014-06-23

**Authors:** Yasuaki Tamaki, Tomohiro Goto, Daisuke Hamada, Toshihiko Nishisho, Kiminori Yukata, Suzue Naoto, Hiroshi Egawa, Koichi Sairyo

**Affiliations:** Department of Orthopedics, Institute of Health Biosciences, The University of Tokushima Graduate School, 3-18-15 Kuramoto, Tokushima 770-8503, Japan

## Abstract

The surface finish of a femoral stem plays an important role in the longevity of cemented total hip arthroplasty. In efforts to decrease the rate of aseptic loosening, some prostheses have been designed to have a roughened surface that enhances bonding between the prosthesis and cement, but clinical outcomes remain controversial. We present a rare case of massive osteolysis with extreme femoral expansion that developed after cemented revision total hip arthroplasty. The destructive changes in the femur were attributable to abnormal motion of the stem and were aggravated by the roughened precoated surface of the long femoral component. Revision surgery using a total femur prosthesis was performed because there was insufficient remaining bone to fix the new prosthesis. The surgical technique involved wrapping polypropylene meshes around the prosthesis to create an insertion for the soft tissue, which proved useful for preventing muscular weakness and subsequent dislocation of the hip.

## 1. Introduction

Aseptic loosening is the most common cause of total hip arthroplasty failure and is generally caused by wear debris from the polyethylene cup. As the specific mechanism of cemented stem loosening is the transmittance of stress or shearing force to the bone-cement interface or prosthesis-cement interface, the surface finish of the femoral stem plays an important role in the longevity of cemented total hip arthroplasty (THA) [1–3]. In the 1980s, roughened, textured, or precoated femoral stems were produced to reduce the rate of aseptic loosening by enhancing the bonding between the stem and cement and good clinical outcomes were reported [4, 5]. On the other hand, unexpectedly, early catastrophic failures were reported as a result of cement fractures and debonding of the stem, followed by particle generation due to cement abrasion because of the roughened surface of the stem, resulting in femoral osteolysis [3, 6–11]. Therefore, the effectiveness of using a roughened precoated stem remains controversial. We report here a rare case of massive femoral osteolysis around a cemented roughened long stem.

## 2. Case Report

The patient underwent primary THA for secondary osteoarthritis due to developmental dysplasia of the left hip at the age of 53 years. At the age of 58 years, he sustained a periprosthetic femoral shaft fracture and underwent revision cemented THA, bypassing the fracture site, using a Harris precoat-plus long stem (Zimmer, Warsaw, Indiana). At the age of 76 years, 18 years after the revision surgery, he was referred to our hospital with severe thigh and knee pain after falling and an inability to bear weight. Physical examination revealed that the range of motion of the left hip and knee was completely limited by pain. Compared to the right leg, the left leg was shorter by approximately 11 cm.

**Figure 1:**
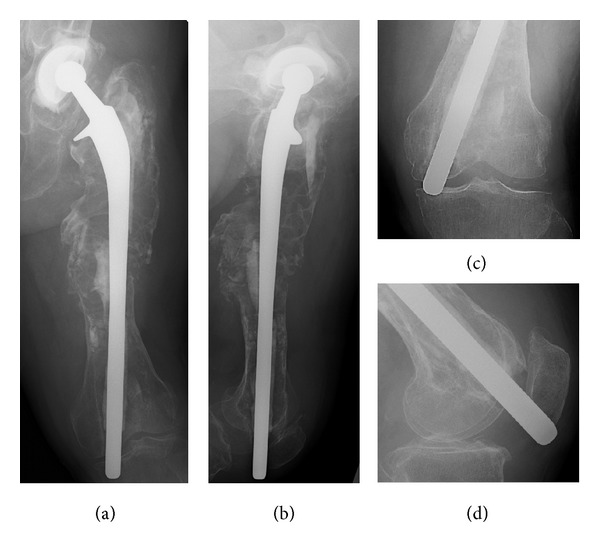
Radiographs of the left femur and knee at initial presentation. Anteroposterior (a) and lateral (b) views of the left femur 18 years after revision surgery show severe loosening of the acetabular cup and femoral stem. Radiographs of the left knee show the distal end of the stem had perforated the medial femoral condyle, reaching subcutaneous tissue through the knee joint (c), (d).

**Figure 2:**
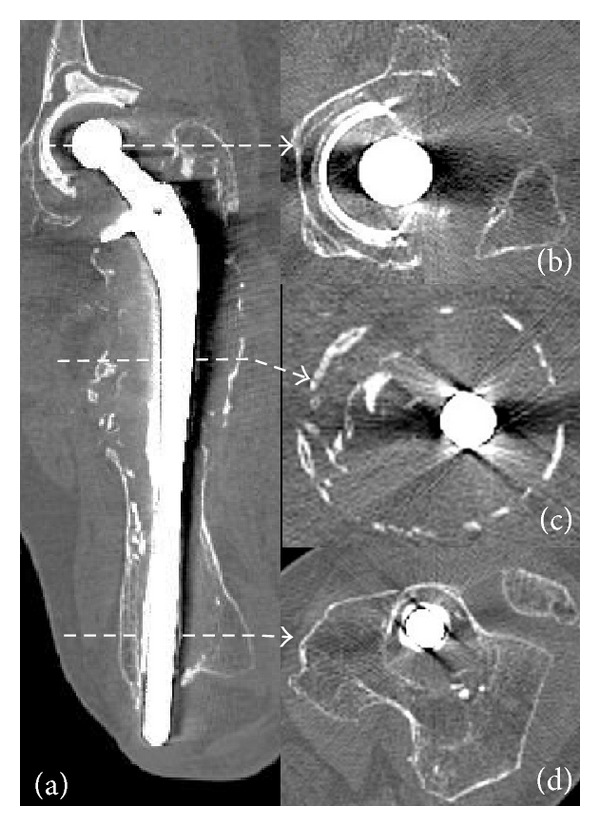
Computed tomography (CT) images of the left femur. Coronal CT image of the left femur shows a massive osteolytic lesion involving two-thirds of the proximal femur with thin cortical fragments surrounding the stem (a). Most of the cement mantle has disappeared except around the greater trochanter and middle of the stem. Axial views from the dotted lines are noted (b)–(d).

Plain radiography of the left femur revealed massive osteolysis involving two-thirds of the proximal femur and perforation of the medial femoral condyle by the distal end of the stem, reaching the subcutaneous tissue through the knee joint (Figures 1(a)–1(d)). Computed tomography revealed marked expansion of the proximal femoral medullary canal, which was surrounded by a thin eggshell-like cortex (Figures 2(a)–2(d)). Cement fracture, debonding of the femoral long stem from the cement mantle, and nonunion of the periprosthetic fracture were suspected. There was no evidence of infection on preoperative culture of fluid aspirated from around the implant. Therefore, the diagnosis was aseptic loosening of the cup and stem due to massive osteolysis.

**Figure 3:**
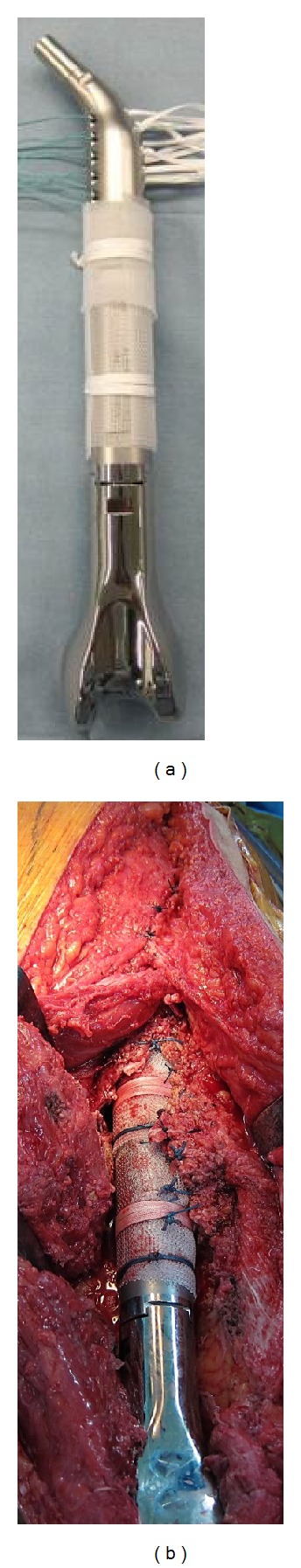
A photograph of the implanted total femur prosthesis (a). Polypropylene meshes wrapped around the prosthesis to create attachment of the surrounding soft tissue (b).

**Figure 4:**
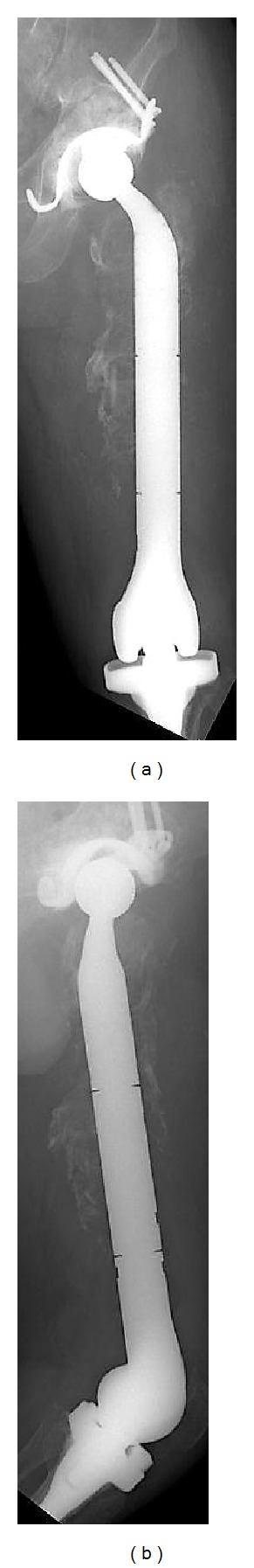
Postoperative radiographs of the left femur showing the total femur prosthesis as implanted. (a) Anteroposterior view. (b) Lateral view.

Reconstructive surgery was performed using a total femur prosthesis (Japan Medical Materials, Osaka, Japan) and a Kerboull-type acetabular reinforcement device (KT-plate; Japan Medical Materials). The patient was positioned in the right lateral decubitus position. We used a posterior approach to the hip, a lateral approach to the femur, and a lateral subvastus approach to the knee joint. The femoral cortex was scattered, and scar tissue filled the expanded medullary canal. The acetabular and femoral prostheses were easily removed because of severe loosening. The surfaces of the acetabular cup and femoral stem had no obvious wear or damage, and there was a small residual cement particle on the surface of the removed stem. The femorotibial joint showed a large osteochondral defect (3 cm in diameter) in the medial femoral condyle, as suggested by radiographic findings (Figure 1). It was difficult to preserve the distal femur, although we could still maintain continuity between the gluteus medius muscle, greater trochanter, and vastus lateralis muscle. Some residual eggshell-like femoral cortex, together with the muscle attachment, was preserved and sutured with a polypropylene mesh wrapped around the total femur prosthesis (Figure 3). Cup reconstruction was performed using a KT-plate with structural bulk and morselized bone allograft (Figure 4).

**Figure 5:**
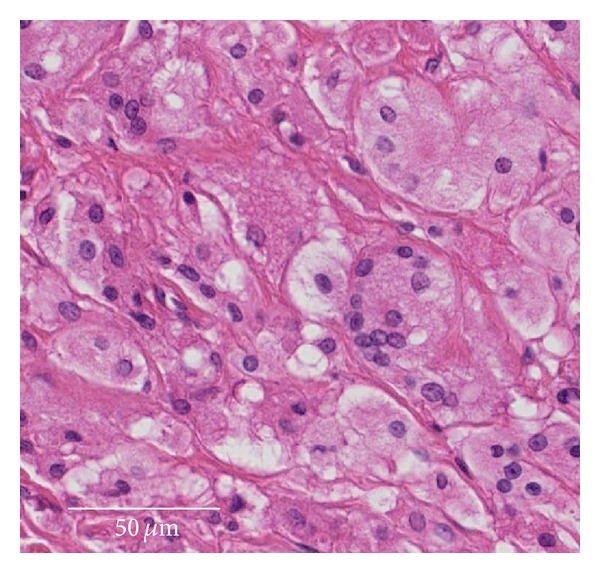
Histologic findings of the scar tissue. There are numerous foamy macrophages but no evidence of acute inflammation. Scale bar: 50 *μ*m.

Histologic findings of the scar tissue from the medullary canal revealed numerous foamy macrophages but no evidence of acute inflammation (Figure 5).

After the final surgery, the postoperative course was uneventful. The patient could perform active knee flexion and extension at 3 days postoperatively and active straight leg raising at 8 days postoperatively. At the time of the latest follow-up, 2 years after the surgery, he was able to walk using a single walking stick and independently perform activities of daily living.

## 3. Discussion

This case is unusual in the extreme feature of massive osteolysis possibly caused by a cemented roughened long stem. To our knowledge, only one similar case has been reported by Kalhor et al. In their case, massive femoral shaft osteolysis was associated with a Wagner cementless long stem, and they suggested that poor initial fit of the stem, stem motion, and fluid pressure induced a pendulum type of stem motion [12]. However, the causes of the massive osteolysis are likely to be different in the two cases because the radiologic shapes of the osteolytic defects were not consistent.

The Harris precoat-plus prosthesis used in our patient has a roughened or textured surface with a proximal methylmethacrylate precoating that is designed to prevent aseptic loosening by improving the bond between the femoral component and cement. Harris et al. have reported excellent long-term results with this system [13–16]; however, other researchers have reported a high rate of aseptic loosening [3, 6–11]. Therefore, the effectiveness of using a roughened precoat has yet to be clarified. The specific mechanism underlying loosening of a cemented rough surface stem is the transmittance of stress or shearing forces to the bone-cement interface because of the rigid bonding between the cement and prosthesis; this can result in early failure. Once loosening occurs, the rough surface of the stem generates a considerable amount of cement debris, which may then lead to osteolysis [11]. In the present case, there was no obvious wearing of the cemented polyethylene cup. Nonunion after the femoral shaft fracture might have created continuous shearing forces, which were applied at the bone-cement interface, cement-stem interface, and cement mantle itself, resulting in early debonding of the stem. Micromotion of the loosened long stem with a large area of rough surface then generated the large amount of cement debris, eventually leading to progressive massive osteolysis [12, 17, 18].

To prevent muscle weakness and subsequent dislocation of the hip, we preserved the residual femoral cortex together with the muscle insertions during surgical reconstruction by suturing these tissues with a polypropylene mesh wrapped around the stem. Polypropylene mesh was originally developed for hernia repair. The technique of using this mesh to anchor the surrounding soft tissue has been applied for several orthopedic indications, with satisfactory clinical results obtained for reconstruction of the patellar tendon [19], chest wall defects following tumor resection [20], and pseudocapsules in arthroplasty [21]. In the present case, this surgical technique minimized loss of function, even though biological reconstruction of the hip abductors and soft tissue reattachment at the proximal of the femur were not achieved. Other surgical options such as impaction of a bone graft together with a strut allograft [22] or an allograft-cemented composite using an interlocking nail stem [23] may be indicated for massive osteolysis. However, these techniques were not suitable in the present case because there was no reliable femoral cortex in two-thirds of the proximal femur and a large defect was present in the medial femoral condyle.

We have presented a unique case of massive osteolysis secondary to loosening of a cemented roughened long stem. Good short-term outcome was achieved using a total femur prosthesis. The combined use of a roughened long stem with cementing carries a risk of massive osteolysis over the long term, although the etiology of the osteolysis in the present case is not completely clear. This case suggests the importance of careful observation and frequent follow-up of patients treated using a cemented roughened long stem.
